# Global infectious disease research collaborations in crises: building capacity and inclusivity through cooperation

**DOI:** 10.1186/s12992-021-00731-2

**Published:** 2021-07-26

**Authors:** Jonathon P. Fanning, Srinivas Murthy, Nchafatso G. Obonyo, J. Kenneth Baillie, Steve Webb, Heidi J. Dalton, John F. Fraser

**Affiliations:** 1 Faculty of Medicine, University of Queensland, Brisbane, Australia; 2 Nuffield Department of Population Health, University of Oxford, Oxford, UK; 3 Critical Care Research Group, The Prince Charles Hospital, 627 Rode Road, Chermside, 4032, Level 3, Clinical Sciences Building, Chermside, Queensland Australia; 4 Department of Paediatrics, Faculty of Medicine, University of British Columbia, Vancouver, Canada; 5 Initiative to Develop African Research Leaders (IDeAL)/KEMRI-Wellcome Trust Research Programme, Kilifi, Kenya; 6 Wellcome Trust Centre for Global Health Research, Imperial College London, London, UK; 7 Roslin Institute, University of Edinburgh, Edinburgh, UK; 8 Intensive Care Unit, Royal Infirmary of Edinburgh, Edinburgh, UK; 9 Australian and New Zealand Intensive Care Research Centre, Monash University, Melbourne, Australia; 10 Inova Fairfax Medical Centre, Heart and Vascular Institute, Falls Church, VA USA

**Keywords:** COVID-19, Infectious diseases, Research collaboration, Research networks, Pandemics

## Abstract

**Background:**

The initial research requirements in pandemics are predictable. But how is it possible to study a disease that is so quickly spreading and to rapidly use that research to inform control and treatment?

**Main body:**

In our view, a dilemma with such wide-reaching impact mandates multi-disciplinary collaborations on a global scale. International research collaboration is the only means to rapidly address these fundamental questions and potentially change the paradigm of data sharing for the benefit of patients throughout the world. International research collaboration presents significant benefits but also barriers that need to be surmounted, especially in low- and middle-income countries.

**Conclusion:**

Facilitating international cooperation, by building capacity in established collaborative platforms and in low- and middle-income countries, is imperative to efficiently answering the priority clinical research questions that can change the trajectory of a pandemic.

## Background

Earth is currently facing one of its most significant public health crises in modern history. Since the first cases of coronavirus disease (COVID-19) were notified in December 2019, the world has progressively struggled with this novel infection, seeking to understand its epidemiology, clinical significance, optimal patient management, and prevention and control strategies to reduce morbidity and mortality, as well as the socio-economic consequences.

## Main text

The initial research requirements in pandemics are predictable. Six weeks after Chinese health authorities announced the discovery of a novel coronavirus, the World Health Organization (WHO) established transmission dynamics, disease severity and immunity, and impact of control and mitigation measures as the main domains of research priorities to guide public health responses to COVID-19 [1]. Previous analysis of the scientific literature on severe acute respiratory syndrome (SARS) and Middle East Respiratory Syndrome (MERS) identified ten major themes of research inquiry: clinical characterisation; prognosis; diagnosis; clinical management; viral pathogenesis; epidemiological characterisation; infection prevention and control/transmission among healthcare workers; susceptibility in the community; psychosocial impacts of disease and social isolation; and aetiology [2]. When the same research themes were applied to identify deficiencies in the understanding of COVID-19, it was clear that, although progress has been made, large knowledge gaps persist and no published data existed within some of the themes [2].

But how is it possible to study a disease that is so quickly spreading and to rapidly use that research to inform prevention, control, and treatment? In our view, a dilemma with such a wide-reaching impact mandates collaboration on a global scale. International research collaboration (IRC) is the best means to rapidly address these fundamental questions and potentially change the paradigm of data sharing, constrained by several ethical, legal, and social challenges in addition to technical (data quality, harmonization) and organizational hurdles, for the benefit of patients worldwide. Although several studies on IRC in the context of COVID-19 pandemic have been published, including calls to foster IRC, little is known about their effective impact. We therefore present an overview of IRC in COVID-19 including several examples and describing their scope and impact. We also discuss the benefits and challenges of IRCs in general and in the context of the pandemic, with particular attention to the situation in low and middle-income countries (LMICs).

In recent years, there has been increasing participation in IRCs in various forms, such as multicentre studies, international registries, evidence synthesis, and Delphi consensus studies for the creation of clinical practice guidelines [3]. These enable researchers to share their knowledge and combine perspectives to solve complex cross-disciplinary problems, creating higher impact outputs and contributing to overcome resource fragmentation. The critical mass to scale data generates power to promptly answer pressing clinical questions with generalizability, applicability, and scientific validity across multiple populations [4]. This value has been confirmed across diseases of all incidences and severities, and is possibly even greater in time-critical settings, such as pandemics [5].

Prior pandemics/epidemics, especially the influenza A H1N1 pandemic in 2009, together with major social and technological developments, resulted in the formation of several platforms to facilitate regional and global collaboration in infectious disease research (Table 1). Such consortia leverage scale, multi-disciplinarity, and data sharing to study a pandemic at its various stages in different countries and predict its course. These platforms help to answer critical clinical, public health, and socio-economic questions and provide important public goods.

**Table 1:** Examples of international research consortia launched in recent years in the context of infectious disease response

Acronym	Name of consortium	Consortium composition^a^	COVID-19 research gaps addressed / scope^a^	COVID-19 − related publications^a^
*AFREhealth*	*African Forum for Research and Education in Health*	It is a conglomerate of individuals, institutions, associations and networks from all the geographic and linguistic regions of Africa	Launched the *COVID-19 Research Collaboration on Children and Adolescents* to address existing knowledge gaps. This consortium is expected to generate key evidence to inform clinical practice and public health policymaking for COVID-19, while concurrently addressing other major diseases affecting children in African countries	[6]
*ALERRT*	*African coaLition for Epidemic Research, Response and Training*	Consists of 21 partner organisations from 13 countries (9 African and 4 European)	Multidisciplinary consortium to reduce public health and socio-economic impact of disease outbreaks in sub-Saharan Africa by building sustainable patient-centred clinical and laboratory research preparedness and response network.	[7]
*CCCC*	*COVID-19 Critical Care Consortium*	Over 380 hospitals and affiliated research facilities in 54 countries	World-first database building an ICU profile of COVID-19 patients, applying AI and big data techniques to draw insights from anonymized data.	[8]
*Discovery*	*Discovery, the Critical Care Research Network*	Over 600 hospitals and medical centres worldwide involved in the COVID-19 registry	Creation of a global COVID-19 registry that tracks ICU and hospital care patterns in near real-time	[9]
*ECRAID*	*European Clinical Research Alliance on Infectious Diseases*	Proposed European-wide initiative	Goal of establishing a sustainable clinical research organisation and network for infectious diseases.	[10]
*InFACT*	*International Forum for Acute Care Trialists*	36 national critical care societies, networks, and groups worldwide	Contributing to an effective and coordinated global research response together with the WHO and other consortia.	[5]
*ISARIC*	*International Severe Acute Respiratory and Emerging Infection Consortium*	50 ratified clinical research networks worldwide	Creating tools for investigators to collect and store data in a standardised way and supporting clinical trials of treatments	[11]
*PANDORA*	*Pan-African Network for Rapid Research, Response, Relief and Preparedness for Infectious Diseases Epidemics*	Partners from 13 African institutions and nine European institutions in nine African and four European countries	‘One Health’ initiative that supports broad themes addressing response to emerging infections, including COVID-19, in Africa and supporting this through capacity development and training. It has provided expert advice on SARS-CoV-2 diagnostic tools and validation and developed a COVID-19 diagnostic tool decision-making app	[12]
*PREPARE*	*European Union’s Platform for European Preparedness Against (Re-) Emerging Epidemics*	A common European clinical research infrastructure covering over 600 primary care sites and over 600 hospital sites in 27 EU member States.	Launched the *Rapid European COVID-19 Emergency Research response (RECOVER)* initiative to address urgent questions for patient and public health: citizens’ experience of COVID-19 and its impacts, household transmission and the impact of interventions to mitigate transmission, if and how children may contribute to the spread of the virus, recommendations on protecting the health of those at the frontline	[13]
*ZIKAction*		14 partners across South and Central America, the Caribbean and Europe	A multidisciplinary ready-to-act network capable of addressing any maternal and paediatric research need arising from (re-) emerging infectious diseases	[14]
*ZIKAlliance*		54 partners worldwide coordinated by INSERM, the French National Institute of Health and Medical Research.	Investigates the clinical, fundamental, environmental and social aspects of ZIKA infection and in collaboration with ZikaPLAN and ZIKAction develops the preparedness platform in Latin America and the Caribbean	[15]
*ZikaPLAN*	*Zika Preparedness Latin America Network*	Brings together 25 leading research and public health organizations in Latin America, North America, Africa, Asia, and Europe.	Comprehensive approach to tackling the Zika threat, encompassing epidemiological surveillance, clinical studies, the development of innovative diagnostic tools and control strategies, in addition to education and knowledge-sharing	[16]

Blank cells indicate that publications have not been found or results have not been published yet

*Abbreviations*: *ICU* intensive care unit, *COVID-19* coronavirus disease 2019, *AI* artificial intelligence, *WHO* World Health Organization, *EU* European Union

^a^Information has been obtained from the consortium websites and may not be updated

Moreover, the use of shared research methodologies in diverse locations allows direct comparison between distinct populations, as well as over time. Shared databases encourage data completeness, quality control, harmonization, and facilitates data exchange. Finally, collaborative efforts ensure the synchronization of timelines and avoid competition among studies evaluating similar interventions.

COVID-19 has expanded across every LMIC, where the human impact may be greater. IRCs permit research to be conducted in countries that might otherwise lack the necessary resources and/or expertise. Collaborations of LMICs, so-called ‘South-South’ collaboration, such as the *Africa Taskforce for Coronavirus (AFTCOR)* or the aforementioned *PANDORA*, are often better placed to find solutions to their specific challenges and needs [17]. They also create a critical mass that facilitates cost-effectiveness, improves retention of talented researchers, and increases visibility and participation globally [17]. Many collaborative networks with LMICs also include high-income partners, referred to as ‘North-South’ collaboration [18]. These ‘North-South’ research collaborations previously demonstrated their utility to create global clinical guidelines taking into consideration the evidence base for interventions and the specific challenges of LMICs [3, 19]. In the case of COVID-19, this has facilitated the initiation and ongoing operations of studies within LMICs [8], including increased access to funding, resources, knowledge and experimental treatments with potentially widespread infectious disease control benefits that transcend geographical and socio-economic borders. Moreover, these IRCs also facilitate the investigation of the impacts of socio-cultural biases that might render policies like social distancing or generalised lockdowns untenable and ineffective and help to assess genetic factors that might influence a population’s susceptibility to the virus and treatment responses [18].

Perhaps the greatest advantages of IRCs for LMICs relate to the enhanced dissemination of findings fostering their global impact. In LMICs, traditional routes of scientific data dissemination have had a disproportionately low impact on healthcare practices, due to inadequate resources or decision-making to implement changes and to the reduced access of many healthcare practitioners to high-profile, English-language journals. IRCs involving multiple countries with different levels of affluence facilitate dissemination beyond traditional routes to include more local and open-access journals, newsletters from professional bodies, and media outlets.

Despite the many advantages of IRCs between high- and LMICs, various obstacles need to be overcome. Obstacles include the disproportionate influence of high-income countries on study agendas, which may skew benefits to their favour; ethical issues regarding inconsistency in the acquisition of patient consent, governance of health research, training of human resources, institutionalisation of scientific activity, access to research funds, and cultural aspects; lack of commitment to capacity building; and prioritization of limited resources to healthcare and other essential services in preference to research [20]. However, the mixed ‘patchwork’ of achievements and failures in COVID-19 response may suggest that higher-income nations are not maintaining their commitment to solidarity and equity [21]. Important measures of success of ‘North-South’ collaborations are not limited to scientific advances, but extend to the identification of priority areas of work, ensuring the sustainability of the interventions and investment in local research capacities [20]. The *Global Effort on COVID-19 (GECO) Health Research*, launched in May 2020 by the UK National Institute for Health Research, focuses on understanding the pandemic and mitigating its health impacts in LMIC contexts, encouraging project leadership from LMICs. Twenty projects have been funded until now tackling the consequences of COVID-19 in LMICs, focusing on topics such as transmission and infection control, long-term outcomes, and mental health issues [22, 23]. This and other initiatives attempt to balance discrepant access to research funds between high- and LMICs.

Another major obstacle includes the tremendous increase in the volume and complexity of administrative work and the logistics or contractual requirements for ethical data sharing, which may hinder research productivity. Deficiencies in open-data sharing mechanisms have been reported globally, which may be particularly relevant in LMICs [6, 24]. This highlights the urgent need for interoperable, open-data repositories including real-time deidentified data. Besides, issues with methodological aspects when reporting COVID-19 outcomes and risk factors contribute to increased variability across epidemiological studies [25].

Data sharing within a consortium faces barriers of different nature, as previously discussed. In IRCs, ethical and effective data-sharing can only be achieved by considering the interests of all relevant parties: research participants, researchers, and funders [26]. Researchers in LMICs have shown concerns regarding safeguards when handling data, including transfer to others, and possible lack of control over subsequent data use [27]. Efficient data-sharing frameworks and accountable governance, together with the establishment of institutional data access committees, are urgent requirements to ensure sustainable and fair international data sharing [27].

Data linkage infrastructure, allowing direct download of data from electronic medical records into research databases, changes the paradigm of collaborative medical informatics. Efforts toward the harmonization of data from different IRCs have been successfully implemented, for example in the case of COVID-19 dermatology registries [8, 28]. A recently proposed framework for a data-driven systems approach to the collection, management, and analysis of high-quality data to inform decision in managing clinical responses and social measures to overcome the COVID-19 pandemic and future health crises is currently under development [29].

There are also inter-personal issues, including the development of trust between investigators. The use of pre-existing networks where IRC is already established allows for that trust component to be integrated before a health crisis. IRC also requires funding, which is often subject to a variety of political influences, especially when it crosses borders. One solution to this issue is the creation of multilateral funding organizations, such as the *Global Research Collaboration for Infectious Disease Preparedness (GloPID-R)* that invests in research in new or re-emerging infectious diseases globally [30].

Despite such limitations, numerous international observational and randomised controlled trials are studying COVID-19. The COVID-19 pandemic has rapidly accelerated trends in international collaboration thanks to unique information sharing efforts and mobilization of resources [31, 32]. The pre-existing *Short Period Incidence Study of Severe Acute Respiratory Infections (SPRINT-SARI)* and the recently formed *COVID-19 Critical Care Consortium (CCCC)*, each incorporating more than 350 hospitals in over 48 countries, have activated tiered data collection-based on-site resources, common case report forms, and global dissemination networks to provide observational data in critical care [8, 30, 33]. The *Randomised, Embedded, Multifactorial, Adaptive Platform Trial for Community-Acquired Pneumonia (REMAP-CAP)* is a perpetual multinational trial that simultaneously evaluates interventions across multiple management domains, which can be adapted to evaluate treatment during respiratory pandemics [30]. Several consortia have been created to understand the risks associated with COVID-19 in patients with different comorbidities, such as the *Surveillance Epidemiology of Coronavirus Under Research Exclusion for Inflammatory Bowel Disease* (SECURE-IBD) registry, the *COVID-19 Global Rheumatology Alliance* (COVID-19 GRA), the *European Renal Association COVID-19 Database* (ERACODA), the *Global consortium study of Neurological Dysfunction in COVID-19* (GCS-NeuroCOVID) and the *Thoracic Cancers International COVID-19 Collaboration* (TERAVOLT) [25, 34–38]. Such consortia have also provided crucial data on other topics, ranging from the well-being of healthcare workers during the pandemic to weather and atmospheric factors contributing to COVID-19 transmission and fatality rates [39–41].

One of the best examples of multicentre research studies during the pandemic is the *Solidarity Trial* launched by the WHO. Interim results from the Solidarity Trial, enrolling almost 12,000 patients in more than 400 hospital sites in over 30 countries, revealed that all four treatments evaluated (remdesivir, hydroxychloroquine, lopinavir/ritonavir, and interferon) had little or no effect on overall mortality, initiation of ventilation, and duration of hospital stay in hospitalized COVID-19 patients [42]. *Solidarity II*, a global serologic study for COVID-19 launched by the WHO, will enable standardization of serologic assays worldwide and sharing of scientific protocols, training materials, scientific publications, and survey findings. The Coalition for Epidemic Preparedness Innovations (CEPI), together with the global Vaccine Alliance (Gavi) and the WHO, launched COVAX to ensure equitable access to COVID-19 vaccines.

As the pandemic has spread, the number of centres and investigators willing to work together has also increased, thus extending the reach of the consortium’s impact and the potential positive repercussions for the future. Conversely, new collaborative initiatives continue to emerge and calls for research cooperation with established platforms have been met with variable enthusiasm. By combining efforts, we can improve research efficiency and minimise the burden on participating centres. This work demonstrates the promptness and utility of IRCs during COVID-19, but also highlights several barriers that still need to be overcome to maximize their global benefit. Among them, issues regarding data sharing, quality control, and harmonization; ethical issues; and disparities between high income countries and LMICs emerge as the main obstacles for the success of IRCs during the COVID-19 pandemic.

## Conclusions

Although the race for treatment and prevention continues, there is no doubt that research collaborations are driving COVID-19 knowledge and response. Facilitating IRC in infectious disease and building capacity in established collaborative platforms are imperative to efficiently answer the priority clinical research questions that can change the trajectory of a pandemic. Several obstacles still need to be overcome to maximize the global benefit of IRCs during the COVID-19 pandemic.

## Data Availability

Not applicable.

## Abbreviations

AFREhealth: African Forum for Research and Education in Health
AFTCOR: Africa Taskforce for Coronavirus
ALERRT: African coaLition for Epidemic Research, Response and Training
CCCC: COVID-19 Critical Care Research Consortium
CEPI: Coalition for Epidemic Preparedness Innovations
COVID-19: 2019 Coronavirus disease
ECRAID: European Clinical Research Alliance on Infectious Diseases
Gavi: Global Vaccine Alliance
GECO: Global Effort on COVID-19
GloPID-R: Global Research Collaboration for Infectious Disease Preparedness
IRC: International research collaboration
InFACT: International Forum for Acute Care Trialists
ISARIC: International Severe Acute Respiratory and Emerging Infection Consortium
LMIC: low and middle-income country
MERS: Middle East Respiratory Virus
PANDORA: Pan-African Network for Rapid Research, Response, Relief and Preparedness for Infectious Diseases Epidemics
PREPARE: European Union’s Platform for European Preparedness Against (Re-) Emerging Epidemics
SARS: Severe Acute Respiratory Virus Syndrome
SPRINT-SARI: Short Period Incidence Study of Severe Acute Respiratory Infections
WHO: World Health Organisation
ZikaPLAN: Zika Preparedness Latin America Network
